# The effects of silver nanoparticles on intact wastewater biofilms

**DOI:** 10.3389/fmicb.2015.00680

**Published:** 2015-07-06

**Authors:** Zhiya Sheng, Joy D. Van Nostrand, Jizhong Zhou, Yang Liu

**Affiliations:** ^1^Department of Civil and Environmental Engineering, University of AlbertaEdmonton, AB, Canada; ^2^Institute for Environmental Genomics and Department of Microbiology and Plant Biology, The University of OklahomaNorman, OK, USA

**Keywords:** silver nanoparticles (Ag-NPs), wastewater biofilms, GeoChip, antibacterial effects, functional stability

## Abstract

Silver nanoparticles (Ag-NPs) have strong antibacterial properties, which may adversely affect biological wastewater treatment processes. To determine the overall effect, intact biofilm samples were collected from the rotating biological contactor at the local wastewater treatment plant and treated with 200 mg Ag/L Ag-NPs for 24 h. The biofilm uptake of Ag-NPs was monitored with transmission electron microscopy. Forty-five minutes after Ag-NP application, Ag-NPs were seen in the biofilm extracellular polymeric substances (EPS). After 24 h, Ag-NPs had entered certain microbial cells, while other cells contained no observable Ag-NPs. Some cells were dying after the uptake of Ag-NPs. However, there was no significant reduction in cultivable bacteria in the biofilms, based on heterotrophic plate counts (HPC). While this may indicate that wastewater biofilms are highly resistant to Ag-NPs, the HPC represents only a small portion of the total microbial population. To further investigate the effects of Ag-NPs, a GeoChip microarray was used to directly detect changes in the functional gene structure of the microbial community in the biofilm. A clear decrease (34.6% decreases in gene number) in gene diversity was evident in the GeoChip analysis. However, the complete loss of any specific gene was rare. Most gene families present in both treated and untreated biofilms. However, this doesn’t necessarily mean that there was no change in these families. Signal intensity decreased in certain variants in each family while other variants increased to compensate the effects of Ag-NPs. The results indicate that Ag-NP treatment decreased microbial community diversity but did not significantly affect the microbial community function. This provides direct evidence for the functional redundancy of microbial community in engineered ecosystems such as wastewater biofilms.

## Introduction

By October 2013, there were 383 consumer products containing nano-silver, making nano-silver the most commonly used nanomaterial in consumer products for over 5 years (The Project on Emerging Nanotechnologies, 2014). It is inevitable that silver nanoparticles (Ag-NPs) will be released into domestic and industrial waste streams (Benn and Westerhoff, 2008; Hagendorfer et al., 2010) considering the high rate of use. These Ag-NPs could potentially cause adverse effects on microbial communities in biological wastewater treatment systems due to their antimicrobial properties. Considerable attention has been paid to this since the boom in application of nano-silver in consumer products, particularly after 2010. However, this research has been limited to specific groups of microbes and most of the studies have been done on either pure cultures or lab-scale activated sludge systems. In addition, the characterization of microbial communities affected by Ag-NPs in biological wastewater treatment systems has not been directly linked to community function due to a lack of environmental sample sequence annotation in existing databases. Therefore, the effects of Ag-NPs on functional genes in biological wastewater treatment systems need to be monitored directly, especially the effects on wastewater biofilms, which have limited information discovered so far.

Comparatively speaking, inhibition of nitrification by Ag-NPs has been relatively well-characterized in previous research. It is well-accepted that nitrification can be inhibited by Ag-NPs even at concentrations lower than 1 mg Ag/L; the effects are dependent on the dose, the particle size, and the coating of Ag-NPs (Choi and Hu, 2008, 2009; Nguyen et al., 2012; Yuan et al., 2013). The mechanism of Ag-NP toxicity involves membrane disruption, gene expression, enzyme inhibition, and energy production, and is closely associated with silver dissolution (Radniecki et al., 2011; Arnaout and Gunsch, 2012; Yuan et al., 2013). Ammonia oxidizing bacteria (AOB) are more sensitive to Ag-NPs than nitrite oxidizing bacteria (NOB), (Yang et al., 2013, 2014a). An elevated ammonia concentration can increase the toxicity of Ag-NPs (Mumper et al., 2013), while greater water hardness decreases toxicity (Yang et al., 2013; Anderson et al., 2014). At sublethal concentrations, Ag-NPs can upregulate *amoA* (Yang et al., 2013). A few reports have documented the adverse effects of Ag-NPs on nutrient removal from wastewater are dose dependent and nutrient removal can recover with time (Chen et al., 2013; Alito and Gunsch, 2014; Jeong et al., 2014). The effects of Ag-NPs on other microbial functional groups in biological wastewater treatment systems are far less well studied.

Biofilms are commonly used in a relatively large proportion of current biological wastewater treatment systems, such as rotating biological contactors (RBCs) and trickling filters. Previous studies have shown that microbial biofilms are more tolerant to antimicrobial agents than planktonic bacteria (Liu et al., 2007; Sheng and Liu, 2011). However, most of the current research has focused on pure-cultured planktonic or activated sludge systems under controlled conditions in the lab, and it is well recognized that full-scale systems are much more complicated than the scaled-down laboratory experimental systems (Wong et al., 2005). Moreover, it has recently been reported that sulfidation plays an important role on the fate of Ag-NPs in wastewater treatment systems and can significantly reduce Ag-NP toxicity, since sulfide concentration can be high in the wastewater treatment process, especially under anaerobic conditions (Hedberg et al., 2014; Kent et al., 2014; Liu et al., 2014). It is estimated that microgram per liter concentrations of nano-silver may be reaching wastewater treatment plants in North America (Gottschalk et al., 2010; O’Brien and Cummins, 2010; Tugulea et al., 2014), yet the effective concentration (the concentration actually resulting in toxicity) of Ag-NPs in wastewater treatment plants is likely lower than this estimation due to sulfidation. Therefore, to set regulation limits, it is essential to determine the real-world impacts of Ag-NPs on biofilms in wastewater treatment plants.

While some pyrosequencing of Ag-NP-exposed biological wastewater treatment system microbial communities have been performed (Yang et al., 2014b), direct information on the functional structure of microbial community is lacking. It has also been suggested that microbial communities in complicated ecosystems are functionally redundant (Lawton and Brown, 1993; Yin et al., 2000; Briones and Raskin, 2003). GeoChip analysis makes it possible to carry out systematic studies on the microbial community in terms of functional potential. In addition, microarrays interrogate samples against the exact same probe set, so as long as the appropriate probe set is present, low abundance populations are less likely to be missed. GeoChip 4 contains over 82,000 probes targeting 410 functional gene families (141,995 coding sequences), and covers genes associated with carbon, nitrogen, and sulfur cycling, phosphorus utilization, antibiotic and metal resistance, fungi function, etc. (Lu et al., 2012; Tu et al., 2014).

In this study, intact wastewater biofilms from a local wastewater treatment plant were treated with Ag-NPs. Tests were performed in wastewater from the plant to provide the same pH, ionic strength, and natural organic matter present in the plant. Transmission electron microscopy (TEM) was used to examine the biofilm uptake of Ag-NPs. GeoChip analysis was done to investigate the effects of Ag-NPs on the functional structure of the microbial community in the biofilm. The abundance of functional genes in 12 categories was monitored. Functional redundancy and its role in the tolerance of wastewater biofilms to Ag-NPs are discussed.

## Materials and Methods

### Wastewater Biofilm Samples

Wastewater biofilms were collected from the first stage RBC unit in the Devon Wastewater Treatment Plant located in Devon, AB, Canada. The total surface area of the first stage RBC unit is 9290 m^2^. The average daily influent flow is about 2500 m^3^, with an average influent biochemical oxygen demand (BOD) of 157.5 mg/L. All RBC units are run indoors under ambient light. The year-round average room temperature is 20°C, and the water temperature varies from 10–16°C. The average biofilm thickness was 1.5 mm. Biofilms were sampled by cutting out a section (1.5 cm × 1.5 cm, attached to substratum) of the biofilm and substratum just before each experiment. Samples were stored in a Petri dish on ice during transport, and then processed within 30 min of arrival at the laboratory.

### Preparation of Ag-NP Suspensions

Self-dispersing silver nanopowder was purchased from SkySpring Nanomaterials, Inc. (Houston, TX, USA). According to the product description, the Ag-NPs are less than 15 nm, and the particle composition is 10% silver (99.99% purity) and 90% polyvinylpyrrolidone (PVP), similar to Ag-NPs commonly used in commercial products. An Ag-NP suspension of 200 mg Ag/L was prepared by dispersing Ag-NPs in filtered (0.22 μm) wastewater and vortexing for 30 s at the maximum speed.

### Ag-NP Treatment

For each experiment, replicate biofilms were each placed in either 5 mL of filtered wastewater or a Ag-NP suspension and then incubated with shaking (100 rpm) for 24 h in the dark at room temperature (25.5°C). For TEM imaging, the biofilm was sampled by cutting out small sections (0.5 cm × 1.5 cm, attached to substratum) at 0 min (before exposure to Ag-NP), 45 min and 24 h. For cell enumeration and DNA extraction, biofilm was scraped off the RBC substratum after the 24 h incubation. Each experiment was done in triplicate.

### Bacterial Enumeration using Heterotrophic Plate Counts (HPC)

Bacterial enumeration was performed by heterotrophic plate counts (HPC) using the drop plate method (Zelver et al., 1999; Liu et al., 2007). A series of 10-fold dilutions were performed and 10 μL of each dilution was plated on R2A agar in triplicate. Plates were incubated at 31°C for 24 h and held at room temperature for 3 days. Counting was performed with a lower detection limit of 10^2^ CFU/mL. The result was converted into CFU/cm^2^ based on the area of each biofilm sample. *t*-tests were performed in Microsoft Excel 2007 to examine the statistical significance of the results, and corresponding *p*-values were calculated using a type 3 two-tailed *t*-test (unequal SD). A *p*-value less than 0.05 indicated a statistically significant difference.

### TEM Imaging

Transmission electron microscopy samples were prepared using the method described by Palestrant et al. (2004) and Fabrega et al. (2009b). Biofilm samples were fixed immediately after sampling with 2.5% glutaraldehyde in phosphate buffer for 30 min and rinsed with the same buffer three times for 5 min each. Samples were then fixed with 1% OsO_4_ in phosphate buffer for 30 min and rinsed briefly with distilled water, followed by staining with 1% uranyl acetate and dehydrated in a series of ethanol solutions (50, 70, 90, and 100%) for 5 min each. After two more additional changes in 100% ethanol, the samples were embedded in epoxy resin and polymerized at 60°C for 24 h. Polymerized resin blocks were sectioned into ∼60 nm slices and post-stained with uranyl acetate and lead citrate. Samples were visualized using a Philips/FEI (Morgagni) transmission electron microscope with a Gatan digital camera.

### GeoChip Analysis

The Powersoil^®^ DNA Isolation Kit from MO BIO Laboratories, Inc. (Carlsbad, CA, USA) was used to extract genomic DNA from each sample. DNA extracted from the triplicates under each condition (with/without Ag-NPs) were pooled, respectively. Pooled DNA (1 μg)was labeled with Cy3 and hybridized to the GeoChip 4 microarray synthesized by NimbleGen (Madison, WI, USA) and processed as previously described by Lu et al. (2012). The signal-to-noise ratio threshold for a spot to be considered positive was ≥2 as described previously (He et al., 2010). Pearson’s correlation coefficient (*r*) was calculated as a measure of the similarity between selected gene profiles (Pearson, 1896). That is, for two profiles of normalized gene signal intensity: *X* = {*x_i_* : *i* = 1,…, *n*} for no treatment control and *Y* = {*y_i_* : *i* = 1,…, *n*} for Ag-NP treated sample,

(1) $$r=\frac{\overset{n}{\underset{i=1}{\sum }}\left(x_{i}-\overset{¯}{x}\right)(y_{i}-\overset{¯}{y})}{\sqrt{\overset{n}{\underset{i=1}{\sum }}{\left(x_{i}-\overset{¯}{x}\right)}^{2}.\overset{n}{\underset{i=1}{\sum }}{\left(y-\overset{¯}{y}\right)}^{2}}},$$

where $\overset{¯}{x}=1/n\,\;\sum_{i=1}^{n}\,\;x_{i}\;$ and $\overset{¯}{y}=1/n\,\;\sum_{i=1}^{n}\,\;y_{i}\;\;\;\;\;$

### qPCR Analysis

qPCR was used to quantify total bacteria and bacteria associated with nitrification and denitrification. A CFX 96 real-time PCR system with a C1000 Thermal cycler (Bio-Rad Laboratories, Inc.) was used to run the reactions. 10 μL of SsoFast EvaGreen Supermix (Bio-Rad Laboratories, Inc.), 10 pmol of each primer, 6 μL of sterile water, and 2 μL of DNA template (7 μL of sterile water, and 1 μL of DNA template for total bacteria) were added to each 20 μL reaction system. Primers used and reaction programs are shown in **Table 1** Calibration was performed with serial dilutions of a known quantity of the target fragments. Triplicate reactions were run for all samples analyzed. Melting curves were examined to eliminate primer dimer formation or non-specific amplification.

**Table 1:** qPCR primers and conditions.

Target	Primers	Program^∗^	Reference
Total bacteria	341f 5′-CCTACGGGAGGCAGCAG-3′ 907r 5′-CCGTCAATTCCTTTRAGTTT-3′	3 min at 95°C; 35 cycles of 30 s at 94°C, 30 s at 56°C and 30 s at 72°C.	Muyzer et al. (1993)
*amoA* gene	amoA-1F 5′-GGGGTTTCTACTGGTGGT-3′ amoA-2R-TC 5′-CCCCTCTGCAAAGCCTTCTTC-3′	1 min at 95°C; 40 cycles of 5 s 95°C, 20 s at 57°C and 45 s at 72°C	McTavish et al. (1993)
*Nitrospira* sp.	NSR 1113f 5′-CCTGCTTTCAGTTGCTACCG-3′ NSR 1264r 5′-GTTTGCAGCGCTTTGTACCG-3′	3 min at 95°C; 50 cycles of 30 s at 95°C, 60 s at 60°C	Dionisi et al. (2002)
*Nitrobacter* sp.	Nitro 1198f 5′-ACCCCTAGCAAATCTCAAAAAACCG-3′ Nitro 1423r 5′-CTTCACCCCAGTCGCTGACC-3′	3 min at 95°C; 50 cycles of 20 s at 94°C, 60 s at 58°C and 40 s at 72°C	Graham et al. (2007)
*narG* gene	narG 1960m2f 5′-TAYGTSGGGCAGGARAAACTG-3′ narG 2050m2r 5′-CGTAGAAGAAGCTGGTGCTGTT-3′	30 s at 95°C; 35 cycles of 15 s at 95°C, 30 s at 58°C, and 31 s at 72°C	López-Gutiérrez et al. (2004)
*nirS* gene	nirS 1f 5′-TACCACCCSGARCCGCGCGT-3′ nirS 3r 5′-GCCGCCGTCRTGVAGGAA-3′	30 s at 95°C; 30 cycles of 15 s at 95°C, 20 s at 60°C, and 31 s at 72°C	Braker et al. (1998)
*nirK* gene	nirK 876 5′-ATYGGCGGVCAYGGCGA-3′ nirK 1040 5′-GCCTCGATCAGRTTRTGGTT-3′	30 s at 95°C; 30 cycles of 15 s at 95°C, 30 s at 58°C, and 31 s at 72°C	Henry et al. (2004)
*nosZ* gene	nosZ 2f 5′-CGCRACGGCAASAAGGTSMSSGT-3′ nosZ 2r 5′-CAKRTGCAKSGCRTGGCAGAA-3′	30 s at 95°C; 30 cycles of 15 s at 95°C, 30 s at 60°C, and 31 s at 72°C	Henry et al. (2006)

*All the programs included and a final melting curve analysis from 65 to 95°C, measuring fluorescence every 0.5°C.

## Results

### Uptake of Ag-NPs into the Biofilm and Cells

**FIGURE 1:**
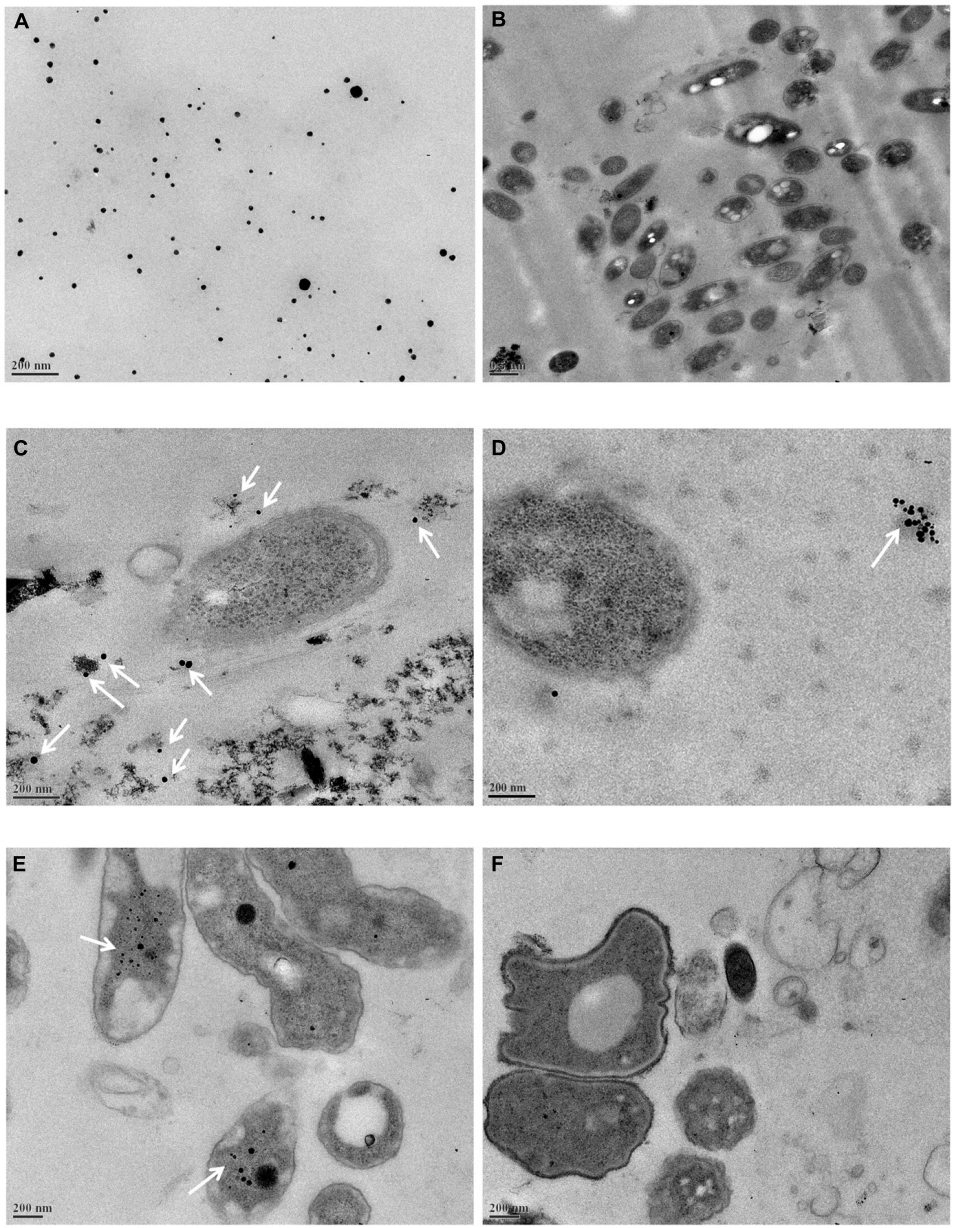
**Transmission electron microscopy (TEM) images. (A)** Silver nanoparticles (Ag-NPs) in wastewater, **(B)** original wastewater biofilms, **(C,D)** wastewater biofilms incubated with Ag-NPs for 45 min, **(E,F)** wastewater biofilms incubated with Ag-NPs for 24 h. Ag-NPs are indicated by white arrows.

Ag-NPs were incorporated into the biofilms quickly after the incubation started. In the abiotic sample in **Figure 1A**, most Ag-NPs are round with a diameter no more than 20 nm and some formed aggregates larger than 50 nm. No particles similar to the Ag-NPs were seen in the control biofilm (**Figure 1B**). After 45 min, Ag-NPs (**Figures 1C,D**, white arrows) were observed in the biofilm and only smaller Ag-NPs entered the biofilms. Over 10 areas were observed in each sample and most of the Ag-NPs were in the biofilm extracellular polymeric substances (EPS) matrix and not in the cells. Some Ag-NPs were near cells (**Figure 1C**), but other Ag-NPs aggregated in the EPS matrix far away from cells. This is consistent with previous research (Holbrook et al., 2006). After 24 h, Ag-NPs were inside some cells and a small fraction of cells with Ag-NPs started to die. Shrinkage and detachment of the plasma membrane from the outer membrane can be seen in **Figure 1E**, which potentially indicates apoptosis (Pandian et al., 2010). Ag-NPs has been reported to cause an apoptosis-like response in bacteria (Lee et al., 2014). However, in over 50% of biofilm areas examined after 24 h of Ag-NP treatment, there were no Ag-NPs observed in the cells, as illustrated in **Figure 1F** There were no significant differences observed between areas near the surface of the biofilm in contact with the bulk liquid and those close to the substratum.

### Overview of the Effects of Ag-NP Treatment on the Biofilm Microbial Community

**FIGURE 2:**
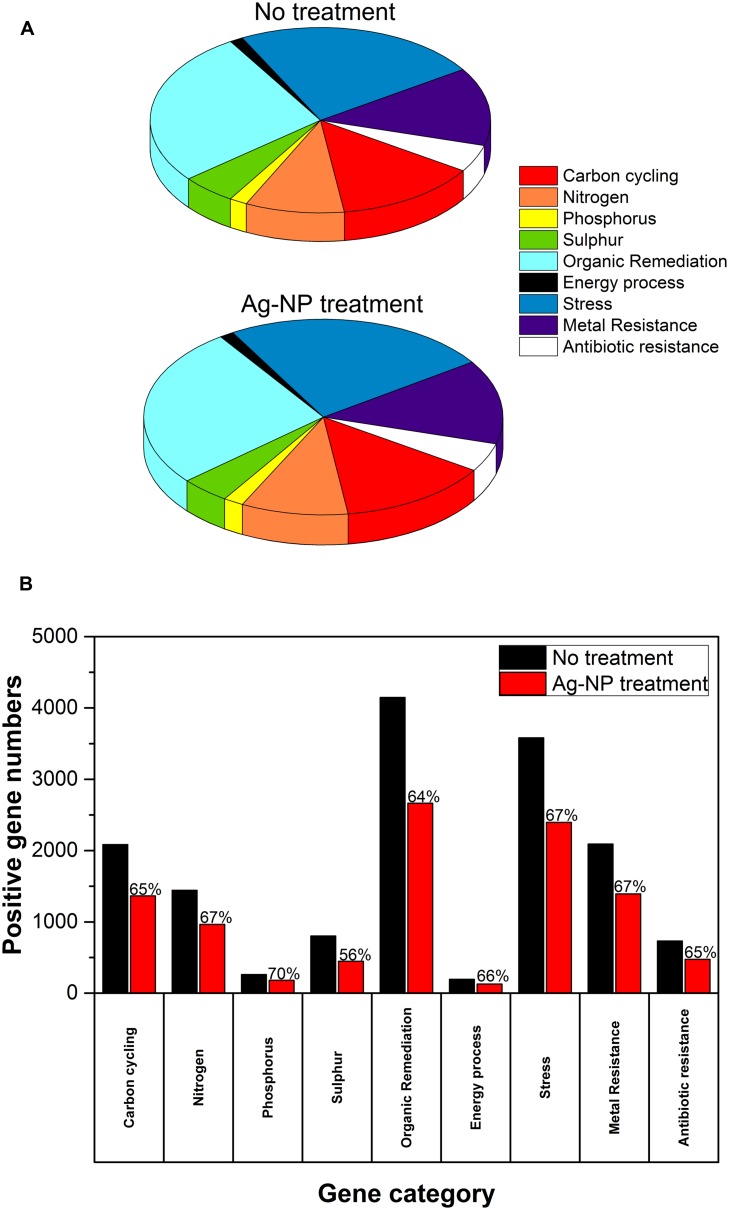
**Effects of Ag-NP treatment on gene abundance.** **(A)** Relative abundance of genes in each category, **(B)** the number of genes detected; the fraction of positive genes detected after Ag-NP treatment is labeled on top of the bar.

After 24 h, the HPC in the wastewater biofilm without Ag-NP treatment was 3.07 × 10^8^ CFU/cm^2^ and the HPC in the wastewater biofilm with Ag-NP treatment was 2.43 × 10^8^ CFU/cm^2^ (**Table 2**). There was no significant change in the viability of heterotrophic bacteria (*p* > 0.05) although the concentration of Ag-NPs applied was as high as 200 mg Ag/L. GeoChip results indicated the relative abundance of genes in each functional category were almost identical with and without Ag-NP treatment as shown in **Figure 2A**, indicating no significant change in evenness (how equal the community is) of the microbial community. Some changes were detected by GeoChip analysis after the 24 h treatment with Ag-NPs. **Figure 2B** shows the number of genes detected (i.e., positive gene number) in each category. A ∼40% decrease in positive gene number was observed with no significant decrease in total signal intensity for each category, indicating that enrichment occurred during the treatment. GeoChip analysis indicated that there was a decrease in richness in the biofilm microbial community after the Ag-NP treatment.

**Table 2:** Viability of heterotrophic bacteria in intact wastewater biofilms under Silver nanoparticle (Ag-NP) treatment.

Sample	Heterotrophic plate counts (HPC) after 24 h (CFU/cm^2^)	*p*-value
No treatment (0 mg Ag/L)	3.07 × 10^8^ ± 4.48 × 10^7^	0.11
With Ag-NPs (200 mg Ag/L)	2.43 × 10^8^ ± 2.72 × 10^7^	

### Effects of Ag-NP Treatment on Nutrient and Pollutant Removal Genes

**FIGURE 3:**
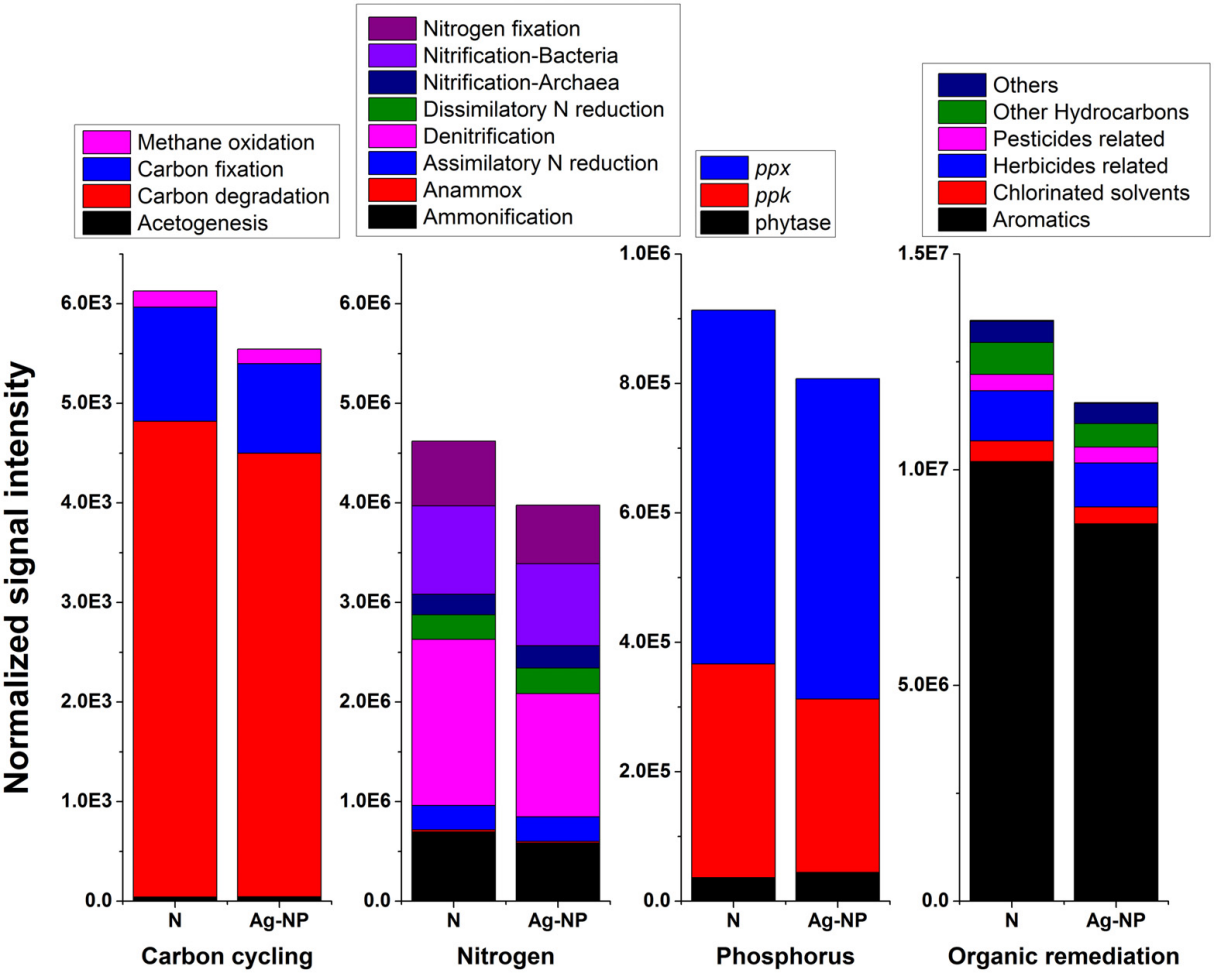
**Effects of Ag-NP treatment on genes associated with nutrient and pollutant removals.** N indicates samples with no treatment and Ag-NP indicates samples with Ag-NP treatment.

Gene profiles associated with carbon cycling, the nitrogen cycle, phosphorus utilization, and organic remediation are illustrated in **Figure 3** There was a slight decrease (<15%) in total signal intensity for these genes. However, in terms of relative abundance of genes in each subcategory (or for specific genes for phosphorus utilization), there was no significant change after Ag-NP treatment. The Pearson correlation coefficients (*r*) between no treatment and Ag-NP treated samples for these four categories were all above 0.99. This indicted that in terms of function, the effects of Ag-NPs were not selective; that is, bacteria are equally sensitive to Ag-NPs if they are considered as functional groups. There was a small proportion of bacteria killed by Ag-NPs in each functional group. However, since the majority of bacteria survived in each functional group, the wastewater biofilm was still capable of degrading numerous types of nutrients and pollutants.

### Response of the Biofilm Microbial Community to Stress Caused by Ag-NPs

At the gene level, the trend was similar to the trends discussed above. There was a slight decrease in signal intensity in each gene but the relative abundance of each gene did not change significantly. However, when the lineage of each gene was examined, differences were observed as discussed below.

#### Response of the Microbial Community to Silver Species

**FIGURE 4:**
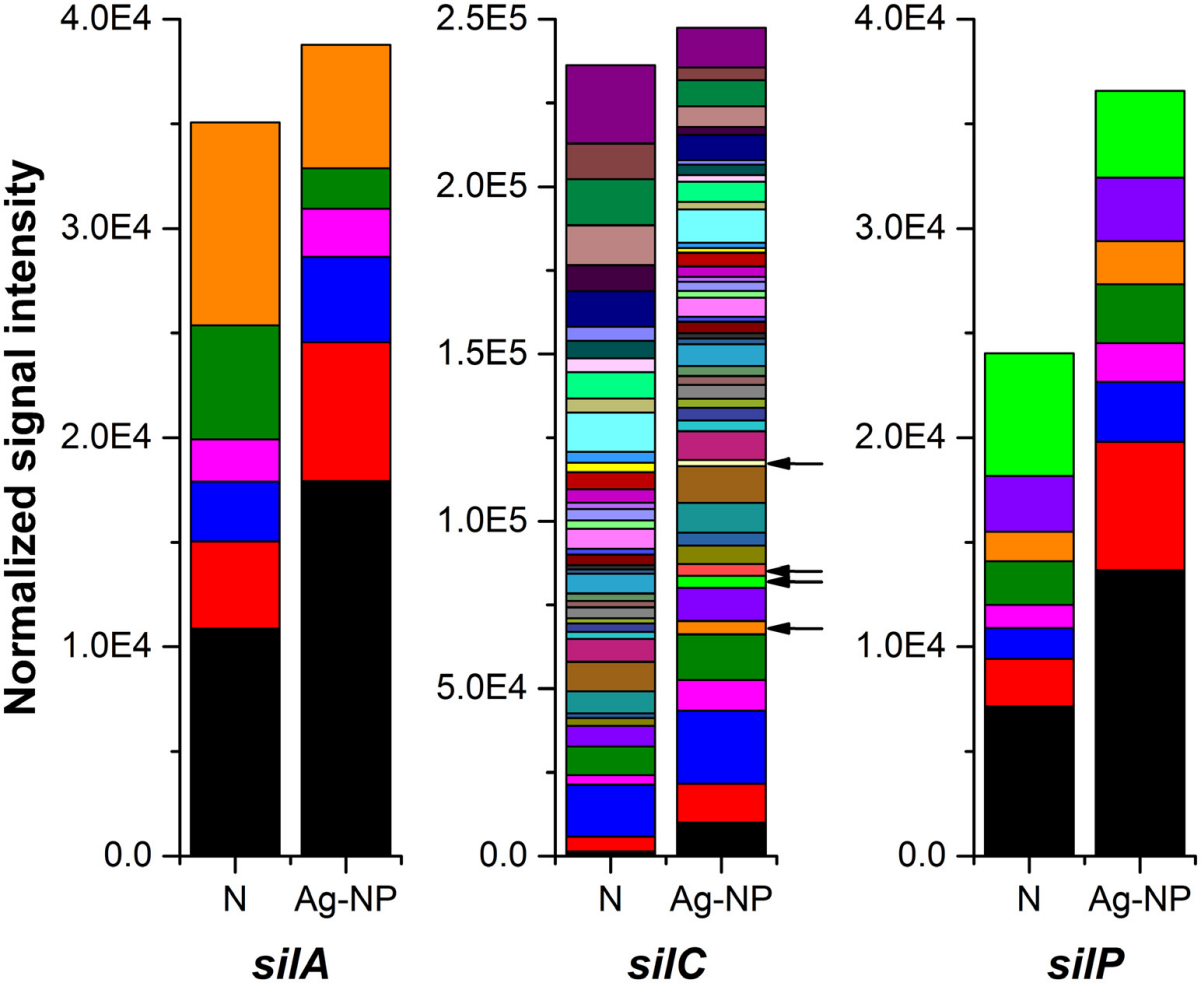
**Effects of Ag-NP treatment on genes associated with silver resistance.** N indicates samples with no treatment and Ag-NP indicates samples with Ag-NP treatment. Colors are coded according to the lineage; genes from gene variants present only in Ag-NP treated samples are indicated with black arrows.

Overall, the total number of silver resistance gene (*silA*, *silC*, *silP*) variants decreased by 34% after Ag-NP treatment while the total signal intensity didn’t decrease as much (only 17%). This trend is similar to genes in other categories: Ag-NP reduced gene diversity but the effect on the corresponding overall function is not as significant. Some gene variants were missing after Ag-NP treatment (listed in Supplementary Table S1). However, this reduction has been compensated by the increase of some other variants. If only genes that were detected after Ag-NP treatment are considered, there was a higher abundance of these genes in the Ag-NP exposed samples (**Figure 4** with the lineage color coded). For *silA* and *silP*, the signal intensity for most of the gene variants (four out of six strains and seven out of eight strains, respectively) increased in the Ag-NP treated sample. For *silC*, a similar increase in abundance was observed as well as some additional gene variants not detected in the control biofilm and indicated by black arrows in **Figure 4** They are derived from *Rhodospirillum rubrum* ATCC 11170, *Pseudomonas syringae* pv. *syringae* B728a, *Burkholderia* sp. H160, and *Ralstonia pickettii* 12J. *Ralstonia pickettii* 12J is a heavy-metal resistant bacterium (NCBI-BioProject, 2008), and the other three all belong to the phylum *Proteobacteria*, which is very common in wastewater treatment systems. While the presence of these four specific strains cannot be absolutely confirmed based on such a short probe, the functional gene derived from or similar to these strains is present. In addition, these results do indicate that some strains are enriched in the Ag-NP treated sample.

#### Response of the Microbial Community to Oxidative Stress

**FIGURE 5:**
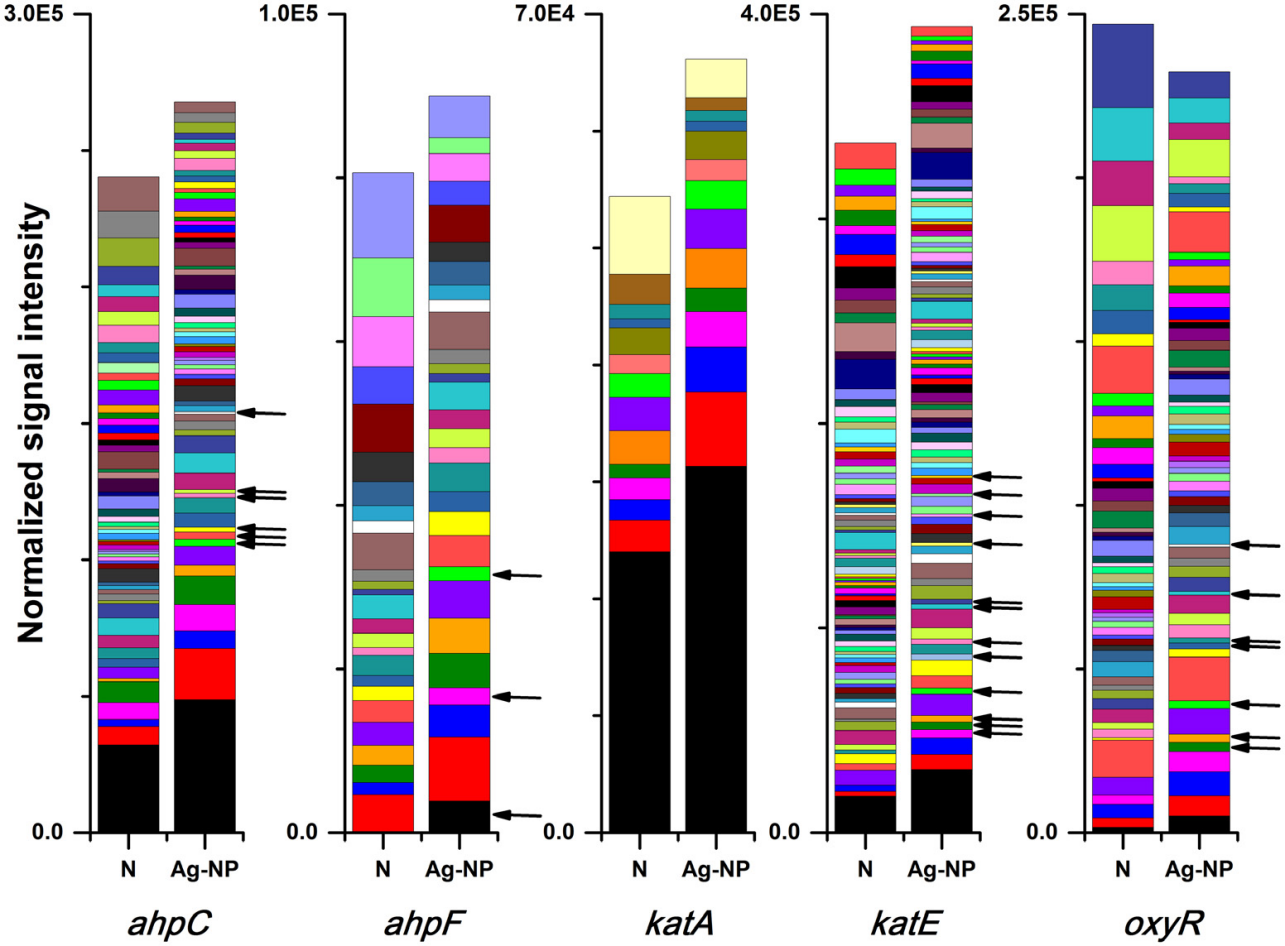
**Effects of Ag-NP treatment on genes associated with oxidative stress.** N indicates samples with no treatment and Ag-NP indicates samples with Ag-NP treatment. Colors are coded according to the lineage; genes from gene variants present only in Ag-NP treated samples are indicated with black arrows.

It is well accepted that one important mechanism for the toxicity of Ag-NPs is oxidative stress caused by producing reactive oxygen species (ROS; Choi and Hu, 2008; Kim and Ryu, 2013; Lee et al., 2014). Five genes associated with oxidative stress were examined: *ahpC*, *ahpF*, *katA*, *katE*, *oxyR*. Genes *ahpC* and *ahpF* encode the two components of the alkyl hydroperoxide reductase. This enzyme detoxifies hydroperoxides produced under oxidative stress (Smillie et al., 1992). Genes *katA* and *katE* encode two kinds of catalases. Gene *katA* is specifically induced by hydrogen peroxide while *katE* encodes a general sigma-factor dependent stress protein (Engelmann and Hecker, 1996). Both alkyl hydroperoxide reductase and the catalases require the positive regulator, *oxyR* gene, for hydrogen peroxide induction, and the *oxyR* gene functions as a positive regulator (Christman et al., 1989; Smillie et al., 1992; Dalla Costa et al., 2009). The response of these genes was very similar to the response of the silver resistance genes. The number of gene variants detected decreased by 36, 34, 42, 36, and 39% for *ahpC*, *ahpF*, *katA*, *katE*, *oxyR*, respectively. Again, the reduction in total signal intensity remained below 20% for most of these genes, indicating more significant reduction of gene diversity instead of overall function. The only exception is the *oxyR* gene, where the total signal intensity decreased by 32%. This unique decrease in the regulator gene *oxyR* may indicates that cells didn’t have much chance to adapt to Ag-NPs before they were inhibited when high concentration of Ag-NP was used. If only strains detected in the Ag-NP treated samples are considered, the majority of these strains increased in abundance in the Ag-NP treated sample (44 out of 66 strains, 20 out of 29 strains, 11 out of 14 strains, 68 out of 101 strains, and 41 out of 63 strains for *ahpC*, *ahpF*, *katA*, *katE*, *oxyR*, respectively). Gene variants that were detected only in the Ag-NP treated sample (**Figure 5**, black arrows) indicated that genes associated with oxidative stress were also enriched in the presence of Ag-NPs. Specifics of gene variants reduced and enriched has been included in the Supplementary Tables S1 and S2.

### Confirmation by qPCR

**FIGURE 6:**
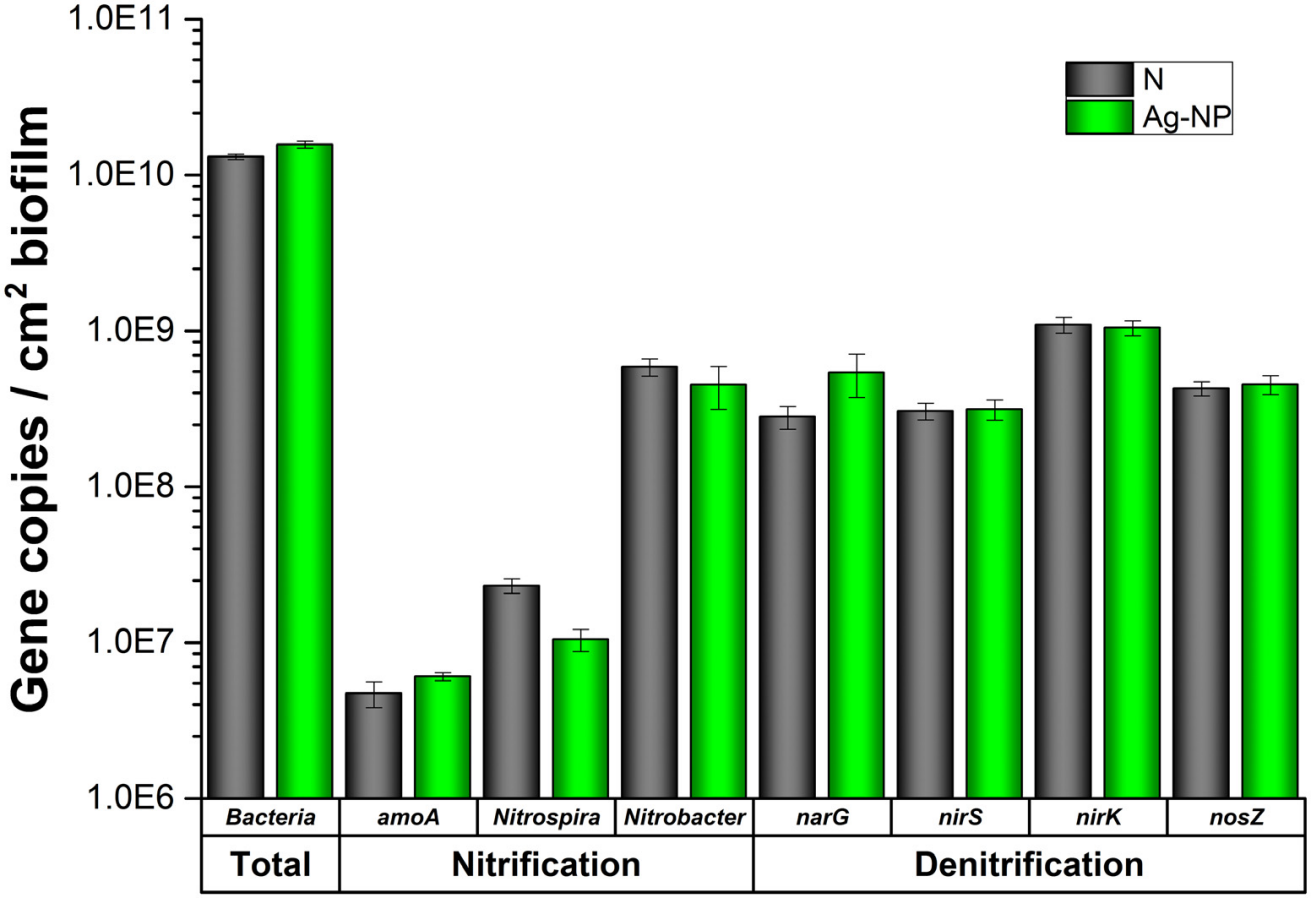
**qPCR results.** N indicates samples with no treatment and Ag-NP indicates samples with Ag-NP treatment. Error bar indicates SD.

The total bacteria was quantified with qPCR to confirm the HPC results, and the number of two major functional groups (nitrification and denitrification) of bacteria was also quantified by qPCR to confirm the GeoChip results. As shown in **Figure 6**, there was no significant difference between the Ag-NP treated biofilm and the control biofilm in terms of total bacteria density. Slightly larger amount of bacteria was detected in the Ag-NP treated biofilm. However, the difference is smaller than one log unit and is not statistically significant. Although cultivable heterotrophic bacteria account for only a small proportion of the total microbial community, this result suggests that qPCR and HPC results are consistent with each other. Wastewater biofilms are highly tolerant to Ag-NPs. For nitrification and denitrification bacteria, there was no significant difference in majority of the genes examined. There was a minor decrease in *Nitrospira* sp. and a minor increase in *narG* gene. However, if the nitrification and denitrification bacteria are considered as two groups, no significant difference was observed. Especially when the ratio of nitrification and denitrification bacteria to the total bacteria was calculated, there was no statistically significant difference. The ratio of nitrification to total bacteria for control and Ag-NP treated biofilms were 5 and 3%, respectively. The ratio of denitrification to total bacteria for control and Ag-| NP treated samples were 16 and 15%, respectively. All the *p*-values are larger than 0.05, indicating a good agreement with GeoChip results showing no significant difference in relative abundance.

## Discussion

### Functional Stability and Functional Redundancy

GeoChip functional gene analysis indicated that wastewater biofilm functions are fairly robust in the presence of Ag-NPs. Our results showed no significant changes in the relative abundance of functional genes in wastewater biofilms after 24 h of Ag-NP treatment at 200 mg Ag/L. This is consistent with previous research (Davies, 2003; Liu et al., 2007; Sheng and Liu, 2011). There was no significant change at the category, subcategory, and gene levels (*r* > 0.99). For each gene, there was loss of signal from certain gene variants, which resulted in a loss of positive gene numbers. However, this loss was always compensated for by either an increased abundance of residual strains or an enrichment of non-dominant strains. It is clear that each functional gene has redundancies from many different strains carrying the same gene and that the loss of a single or even several strains is compensated for by an increase in abundance of other strains. It has long been believed that, in an ecosystem, multiple species can perform similar functions. That is to say that these species are functionally redundant and thus are substitutable with minimal impact on the overall function of the ecosystem (Lawton and Brown, 1993; Rosenfeld, 2002). This functional redundancy has been found in both natural and engineered ecosystems (Briones and Raskin, 2003; Siripong and Rittmann, 2007). Results in this study provided direct evidence for the functional redundancy of microbial communities in engineered ecosystems. However, it should be noted that necessary redundancy is required to ensure the stability of an ecosystem under disturbance (Walker, 1995). The enrichment under Ag-NP treatment led to reduced redundancy in the biofilm microbial community and therefore could lead to a decreased stability under future perturbation, a possibility that needs to be further explored.

### Compositional and Structural Stability

Composition was not as stable as function in the wastewater biofilm microbial community. The decrease in gene number after exposure to Ag-NPs indicated that some bacteria were killed by Ag-NPs, consistent with the TEM observations. The loss and enrichment of genes from different lineages indicated that Ag-NPs triggered changes in the composition of the microbial community. This is consistent with previous research showing that microbial community composition is often sensitive to disturbance (Shade et al., 2012). However, it should be noted that the concentration of Ag-NPs used in this study (200 mg Ag/L) was much higher than what is expected in real wastewater treatment plants (Gottschalk et al., 2010; O’Brien and Cummins, 2010; Tugulea et al., 2014), which is at the microgram per liter range. Two-hundred milligram of Ag/L was chosen according to previous study to make sure detectable changes can be seen (Sheng and Liu, 2011; Sun et al., 2013). However, under such a high concentration of Ag-NPs, the effects of Ag-NPs are still minimal. In addition, the effects of Ag-NPs are dose-dependent (Chen et al., 2012; Nguyen et al., 2012; Sun et al., 2013). Therefore, it is probable that biofilms in wastewater treatment plants will not be significantly affected under current Ag-NP release conditions. A considerable fraction of the Ag-NPs go through aggregation and sulfidation in the EPS matrix and therefore cannot reach microbial cells (Holbrook et al., 2006; Fabrega et al., 2009a; Hedberg et al., 2014; Kent et al., 2014). In addition, no significant change in biofilm structure was observed in the TEM study, indicating that the wastewater biofilms were structurally stable. This structural stability likely contributed to the Ag-NP tolerance of the wastewater biofilm as well. However, it should be noted that the decrease of compositional diversity may make the biofilm more vulnerable to future disturbance and potentially reduce the stability of the system.

## Conflict of Interest Statement

The authors declare that the research was conducted in the absence of any commercial or financial relationships that could be construed as a potential conflict of interest.
