# Supplementary material for: The effects of silver nanoparticles on intact wastewater biofilms
Source: Front Microbiol. 2015 Jul 6;6:680. doi: 10.3389/fmicb.2015.00680 (PMC4491624; doi:10.3389/fmicb.2015.00680)
Supplement: Supplementary file 1 [file Data_Sheet_1.PDF]

The effects of silver nanoparticles on intact wastewater biofilms

Zhiya Sheng [1], Joy D. Van Nostrand [2], Jizhong Zhou [2], Yang Liu [1]*

[1]Department of Civil and Environmental Engineering, University of Alberta, Edmonton, AB, Canada

[2]Institute for Environmental Genomics, Department of Microbiology and Plant Biology, University of Oklahoma, Norman, OK, USA

***Correspondence:** Corresponding Author, Department of Civil and Environmental Engineering, University of Alberta, Edmonton, AB, T6G 2W2, Canada.

yang.liu@ualberta.ca

Keywords: silver nanoparticles (Ag-NPs), wastewater biofilms, GeoChip, antibacterial effects, functional stability.

Supplementary Material

Table S1. Gene variants missing in Ag-NP treated biofilm

Category	Gene	Organisms of missing gene variants
**Silver resistance genes**	*silA*	*Rhodopseudomonas palustris* CGA009
		Sagittula stellata E-37
		Sphingomonas sp. SKA58
		Sulfitobacter sp. NAS-14.1
	silC	*Alcanivorax* sp. DG881
		Alteromonadales bacterium TW-7
		Bordetella petrii DSM 12804
		Burkholderia cenocepacia PC184
		Burkholderia pseudomallei 1655
		Burkholderia ubonensis Bu
		Burkholderia vietnamiensis G4
		Candidatus Desulfococcus oleovorans Hxd3
		Caulobacter crescentus CB15
		Comamonas testosteroni KF-1
		Desulfovibrio vulgaris subsp. *vulgaris* DP4
		Geobacter bemidjiensis Bem
		Geobacter lovleyi SZ
		Gluconacetobacter diazotrophicus PAL5
		Leptothrix cholodnii SP-6
		Methylobacillus flagellatus KT
		Pseudomonas aeruginosa C3719
		Pseudomonas aeruginosa PACS2
		Pseudomonas fluorescens
		Pseudomonas stutzeri A1501
		Ralstonia eutropha H16
		Ralstonia solanacearum UW551
		Rhodoferax ferrireducens DSM 15236
		Salmonella enterica subsp. *enterica* serovar Choleraesuis str. SC-B67
		Sphingomonas sp. SKA58
		Verrucomicrobiae bacterium DG1235
		Xanthomonas campestris pv. *vesicatoria* str. 85-10
	silP	*Bifidobacterium adolescentis* ATCC 15703
		Ralstonia eutropha H16
		Herminiimonas arsenicoxydans
**Genes associated with oxidative stress**	*ahpC*	*Actinomyces urogenitalis* DSM 15434
		Agrobacterium radiobacter K84
		Aspergillus clavatus NRRL 1
		Aspergillus niger
		Bifidobacterium longum NCC2705
		Bordetella avium 197N
		Bordetella parapertussis 12822
		Capnocytophaga ochracea DSM 7271
		Chlamydia muridarum Nigg
		Clavispora lusitaniae ATCC 42720
		Coprinopsis cinerea okayama7#130
		Corynebacterium jeikeium ATCC 43734
		Cyanothece sp. PCC 7425
		Dechloromonas aromatica RCB
		Desulfovibrio magneticus RS-1

Table S1 (continued). Missing gene variants in Ag-NP treated biofilm

Category	Gene	Organisms of missing gene variants
Genes associated with oxidative stress	*ahpC*	*Desulfovibrio vulgaris* subsp. *vulgaris* DP4
		Eggerthella lenta DSM 2243
		Gemmata obscuriglobus UQM 2246
		Geobacter sp. M21
		Gramella forsetii KT0803
		Haliangium ochraceum DSM 14365
		Idiomarina baltica OS145
		Listeria innocua Clip11262
		Marinobacter aquaeolei VT8
		Methylobacillus flagellatus KT
		Mycobacterium abscessus
		Nitrobacter hamburgensis X14
		Nitrobacter winogradskyi Nb-255
		Prosthecochloris vibrioformis DSM 265
		Rhodobacterales bacterium HTCC2654
		Rhodococcus jostii RHA1
		Rhodothermus marinus DSM 4252
		Rothia mucilaginosa ATCC 25296
		Saccharomonospora viridis DSM 43017
		Sphaerobacter thermophilus DSM 20745
		Sphingomonas sp. SKA58
		Stackebrandtia nassauensis DSM 44728
	ahpF	*Alcanivorax borkumensis* SK2
		Bacillus licheniformis DSM 13
		Bacteroides capillosus ATCC 29799
		Brevibacillus brevis NBRC 100599
		Burkholderia thailandensis E264
		Cardiobacterium hominis ATCC 15826
		Desulfobacterium autotrophicum HRM2
		Desulfomicrobium baculatum DSM 4028
		Leuconostoc mesenteroides subsp. *cremoris* ATCC 19254
		Marinobacter algicola DG893
		Nectria haematococca mpVI 77-13-4
		Pantoea sp. At-9b
		Paracoccus denitrificans PD1222
		Pseudomonas putida GB-1
		Teredinibacter turnerae T7901
	katA	*Alicyclobacillus acidocaldarius* LAA1
		Bacillus firmus
		Bacillus subtilis
		Corynebacterium pseudogenitalium ATCC 33035
		Deinococcus radiodurans
		Gluconacetobacter diazotrophicus PAl 5
		Ochrobactrum anthropi ATCC 49188
		Pseudomonas stutzeri A1501
		Pseudomonas syringae pv. *phaseolicola* 1448A
		Rhodococcus opacus B4
	katE	*Acetobacter pasteurianus* IFO 3283-26
		Acinetobacter radioresistens SH164
		Arthrobacter aurescens TC1
		Aspergillus oryzae

Table S1 (continued). Missing gene variants in Ag-NP treated biofilm

Category	Gene	Organisms of missing gene variants
**Genes associated with oxidative stress**	*katE*	*Aurantimonas manganoxydans* SI85-9A1
		Bacillus coagulans 36D1
		Bacillus mycoides DSM 2048
		Bacillus subtilis subsp. *subtilis* str. 168
		Bordetella avium 197N
		Clavibacter michiganensis subsp. *michiganensis* NCPPB 382
		Corynebacterium diphtheriae
		Delftia acidovorans SPH-1
		Desulfovibrio piger ATCC 29098
		Edwardsiella tarda
		endosymbiont of *Onchocerca volvulus*
		Exiguobacterium sp. CNU020
		Frankia alni ACN14a
		Fulvimarina pelagi HTCC2506
		Geobacillus sp. Y412MC10
		Gibberella moniliformis
		Granulibacter bethesdensis CGDNIH1
		Herpetosiphon aurantiacus ATCC 23779
		Lachancea thermotolerans
		Malassezia globosa CBS 7966
		Methanococcoides burtonii DSM 6242
		Methylobacillus flagellatus KT
		Methylocella silvestris BL2
		Mycobacterium avium subsp. *avium* ATCC 25291
		Mycobacterium avium subsp. *paratuberculosis* K-10
		Mycobacterium gilvum PYR-GCK
		Paenibacillus sp. JDR-2
		Paracoccus denitrificans PD1222
		Ralstonia metallidurans CH34
		Rhizobium leguminosarum bv. *trifolii* WSM1325
		Rhodobacter sphaeroides KD131
		Rhodococcus equi
		Rhodococcus erythropolis PR4
		Rhodococcus erythropolis SK121
		Rhodopseudomonas palustris TIE-1
		Roseovarius sp. TM1035
		Schizosaccharomyces japonicus yFS275
		Shewanella putrefaciens 200
		Shewanella sp. ANA-3
		Sinorhizobium meliloti 1021
		Spirosoma linguale DSM 74
		Sporothrix schenckii
		Streptomyces avermitilis MA-4680
		Streptomyces hygroscopicus ATCC 53653
		Uncinocarpus reesii 1704
		Verticillium albo-atrum VaMs.102
		Xanthomonas axonopodis pv. *citri* str. 306
		Yarrowia lipolytica
		Yarrowia lipolytica

Table S1 (continued). Missing gene variants in Ag-NP treated biofilm

Category	Gene	Organisms of missing gene variants
**Genes associated with oxidative stress**	*katE*	*Yersinia mollaretii* ATCC 43969
		Zygosaccharomyces rouxii
		Zymomonas mobilis subsp. *mobilis* NCIMB 11163
	oxyR	*Beijerinckia indica* subsp. *indica* ATCC 9039
		Bradyrhizobium sp. BTAi1
		Congregibacter litoralis KT71
		Corynebacterium urealyticum DSM 7109
		Cronobacter turicensis
		Dinoroseobacter shibae DFL 12
		Frankia sp. EAN1pec
		Geobacillus sp. Y412MC10
		Gluconacetobacter diazotrophicus PAl 5
		Granulibacter bethesdensis CGDNIH1
		Kangiella koreensis DSM 16069
		Legionella drancourtii LLAP12
		Magnetospirillum gryphiswaldense MSR-1
		Maricaulis maris MCS10
		Mycobacterium ulcerans Agy99
		Nitrococcus mobilis Nb-231
		Novosphingobium aromaticivorans DSM 12444
		Ochrobactrum intermedium LMG 3301
		Parvularcula bermudensis HTCC2503
		Pasteurella multocida subsp. *multocida* str. Pm70
		Rhodococcus erythropolis SK121
		Rhodococcus jostii RHA1
		Rhodoferax ferrireducens T118
		Roseovarius sp. HTCC2601
		Ruegeria pomeroyi DSS-3
		Ruegeria sp. R11
		Salinibacter ruber DSM 13855
		Shewanella benthica KT99
		Sinorhizobium meliloti 1021
		Stappia aggregata IAM 12614
		Streptomyces clavuligerus ATCC 27064
		Streptomyces flavogriseus ATCC 33331
		Thiomonas intermedia K12
		Vibrio alginolyticus 12G01
		Vibrio angustum S14
		Vibrio harveyi HY01
		Vibrio mimicus VM223
		Vibrio orientalis CIP 102891
		Vibrio sp. RC586
		Xanthomonas albilineans
		Xanthomonas campestris

Table S2. Gene variants detected only in Ag-NP treated biofilm

Category	Gene	Organisms of missing gene variants
**Silver resistance genes**	*silC*	*Rhodospirillum rubrum* ATCC 11170
		Pseudomonas syringae pv. syringae B728a
		Burkholderia sp. H160
		Ralstonia pickettii 12J
**Genes associated with oxidative stress**	*ahpC*	*Chlorobium phaeobacteroides* DSM 266
		Pseudomonas fluorescens Pf-5
		Dialister invisus DSM 15470
		Actinobacillus succinogenes 130Z
		Lachancea thermotolerans
		Acaryochloris marina MBIC11017
	ahpF	*Magnetospirillum gryphiswaldense* MSR-1
		Bifidobacterium animalis subsp. *lactis* DSM 10140
		Asticcacaulis excentricus CB 48
	katE	*Stigmatella aurantiaca* DW4/3-1
		Bacteroides plebeius DSM 17135
		Streptomyces pristinaespiralis ATCC 25486
		Sphingomonas wittichii RW1
		Methylovorus sp. SIP3-4
		Streptomyces ambofaciens ATCC 23877
		Ajellomyces capsulatus
		Colwellia psychrerythraea 34H
		Roseovarius sp. HTCC2601
		Providencia rettgeri DSM 1131
		Polaromonas naphthalenivorans CJ2
		Parabacteroides johnsonii DSM 18315
	oxyR	*Streptomyces* sp. Mg1
		Streptomyces sp. C
		Erwinia tasmaniensis Et1/99
		Corynebacterium jeikeium K411
		Aliivibrio salmonicida LFI1238
		Capnocytophaga gingivalis ATCC 33624
		Dickeya dadantii Ech703

*Variants in Table S2 are arranged in the same order as indicated by black arrows in Figure 4 and Figure 5 (from bottom to top).
